# Supplementary material for: Pearl millet genomic vulnerability to climate change in West Africa highlights the need for regional collaboration
Source: Nat Commun. 2020 Oct 19;11:5274. doi: 10.1038/s41467-020-19066-4 (PMC7573578; doi:10.1038/s41467-020-19066-4)
Supplement: Supplementary file 1 — Supplementary Information [file 41467_2020_19066_MOESM1_ESM.pdf]

Supplementary Information

Pearl millet genomic vulnerability to climate change in West Africa highlights the need for regional collaboration

Bénédicte Rhoné, Dimitri Defrance, Cécile Berthouly-Salazar, Cédric Mariac, Philippe Cubry, Marie Couderc, Anaïs Dequincey, Aichatou Assoumanne, Ndjido Kane, Benjamin Sultan, Adeline Barnaud, Yves Vigouroux.

Correspondence to: benedicte.rhone@cirad.fr; yves.vigouroux@ird.fr; adeline.barnaud@ird.fr

This PDF file includes:

Supplementary Figures 1 to 12 (Pages 2 to 14)

Supplementary Tables 1 to 3 (Pages 15 to 17)

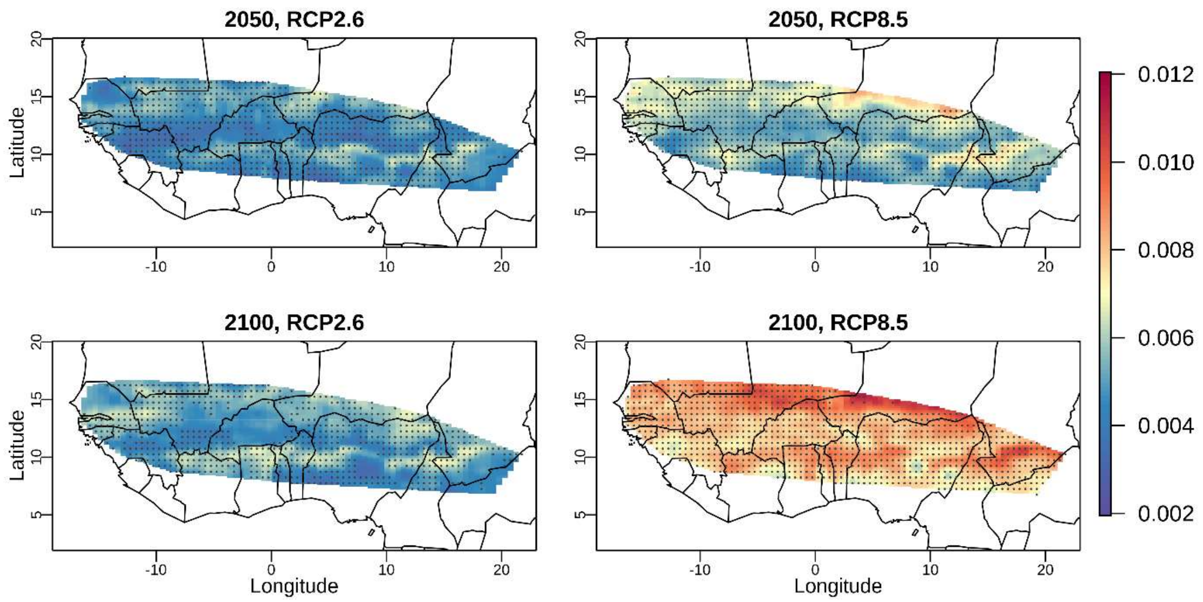

Supplementary Figure 1. **Pearl millet genomic vulnerability at the 2050 and 2100 horizons.**

Genomic vulnerabilities in West Africa were estimated based on projections for two gas concentration pathways, scenarios RCP2.6 and RCP8.5 by 2050 and 2100. Colors ranging from blue to red indicate the mean value of genomic vulnerability estimated for 17 climate model projections. Stippling indicates areas where the magnitude of the mean genomic vulnerability is more than twice the standard deviations (i.e. coefficient of variation < 50%). This indicates regions where the mean genomic vulnerability estimated using multi-model climate projections is consistent across climatic models.

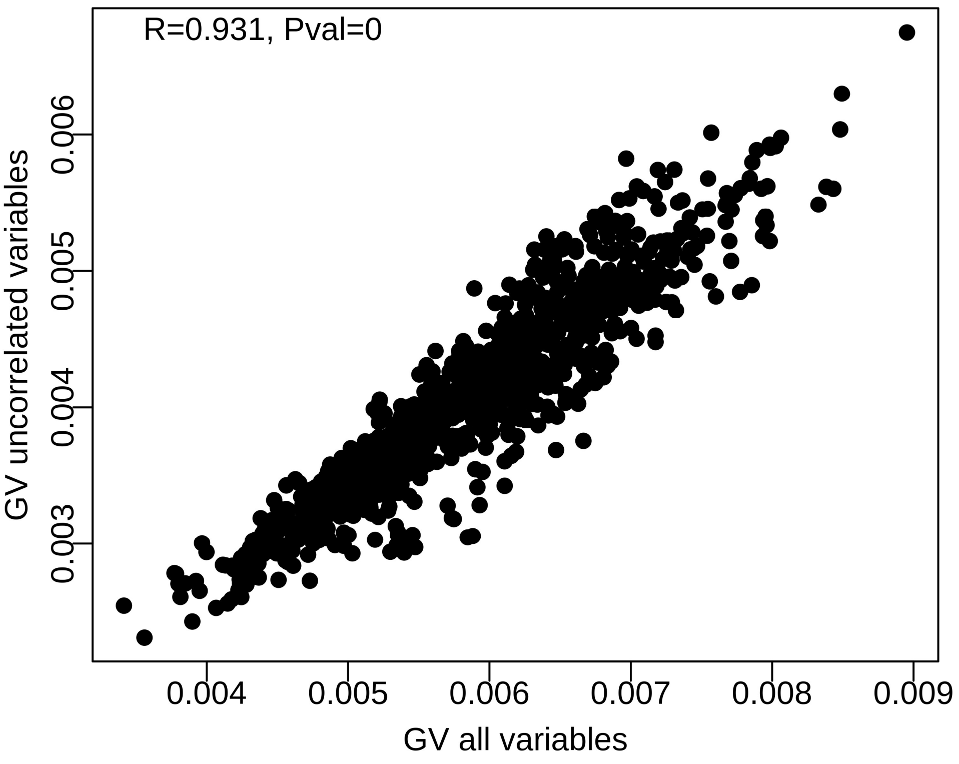

Supplementary Figure 2. **Assessing the impact of correlated climatic variable for the prediction of genomic vulnerability.**

Correlations between genomic vulnerability calculated using all the climate variables and genomic vulnerability calculated only using uncorrelated variables were performed (with a maximum Pearson correlation threshold of 0.7).

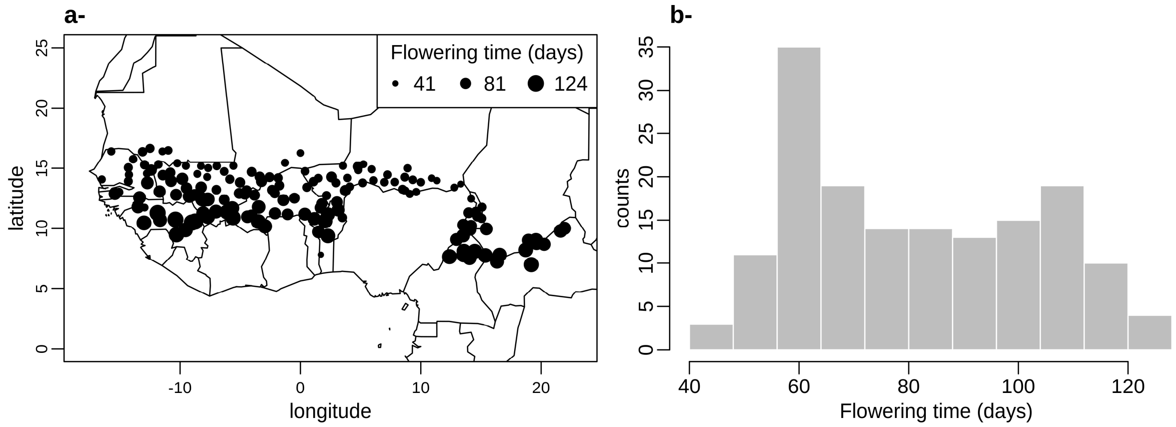

Supplementary Figure 3. **Flowering time of pearl millet landraces.**

Flowering times were assessed in the common garden experiment in Sadoré (Niger). **a** Map of the length of flowering in the landraces sampled in the present study. **b** Distribution of flowering time.

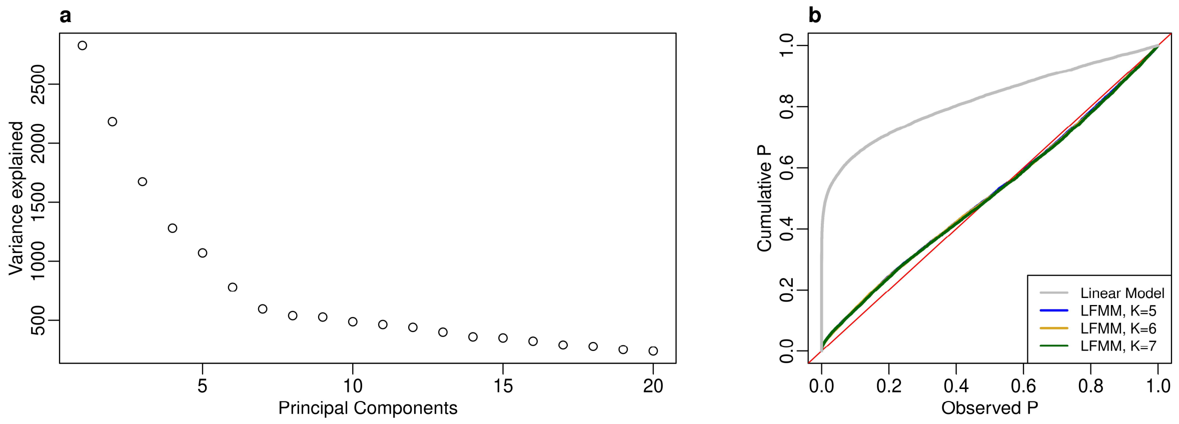

Supplementary Figure 4. **Diagnostic plots for the genomic association analyses with flowering time.**

a Scree-plot of the cumulative variance explained by the axis of the PCA performed on the allele frequencies. We first determine a number of latent factors based on the PCA to perform a genome wide association study (GWAS) using a latent factor mixed model (LFMM). A first inflexion point is observed at K=5, but we also consider K=6 and K=7 for GWAS analysis.

b QQ-plot of the observed and expected *p*-values for the GWAS analyses. We first plot the QQ-plot obtained for the GWAS performed with a simple linear model not taking into account for the confounding genetic structure (linear model in grey). We then plot the QQ-plot obtained for the GWAS analysis for different cofounding factors: K=5 (bleu), K=6 (yellow), K=7 (green).

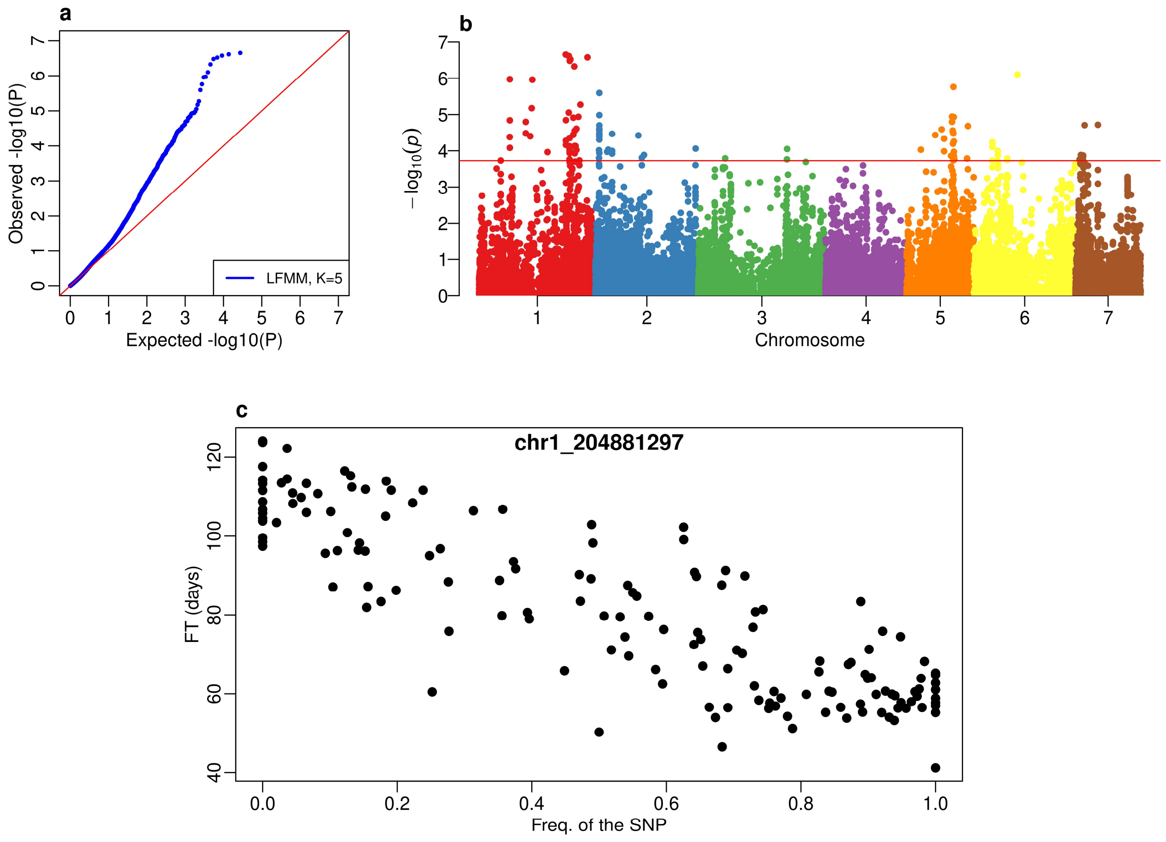

Supplementary Figure 5. **Genome wide analysis for flowering time.**

a The analysis is perform for K=5 using latent factor mixed model (LFMM). The QQ-plot of logarithm of the p-value (–$\log_{10}(P)$) is reported.

b The manhattan plot along the seven chromosomes of pearl millet highlights the pics of interest, grouping the SNPs associated with flowering time. The red line indicates the 5% FDR threshold.

c For the most significant SNP (SNP on chromosome at position 20881297, chr1_20881297, P=2.10^{-7}), a nice correlation is observed between the mean flowering time (FT) of varieties and the allele frequencies for this specific SNP.

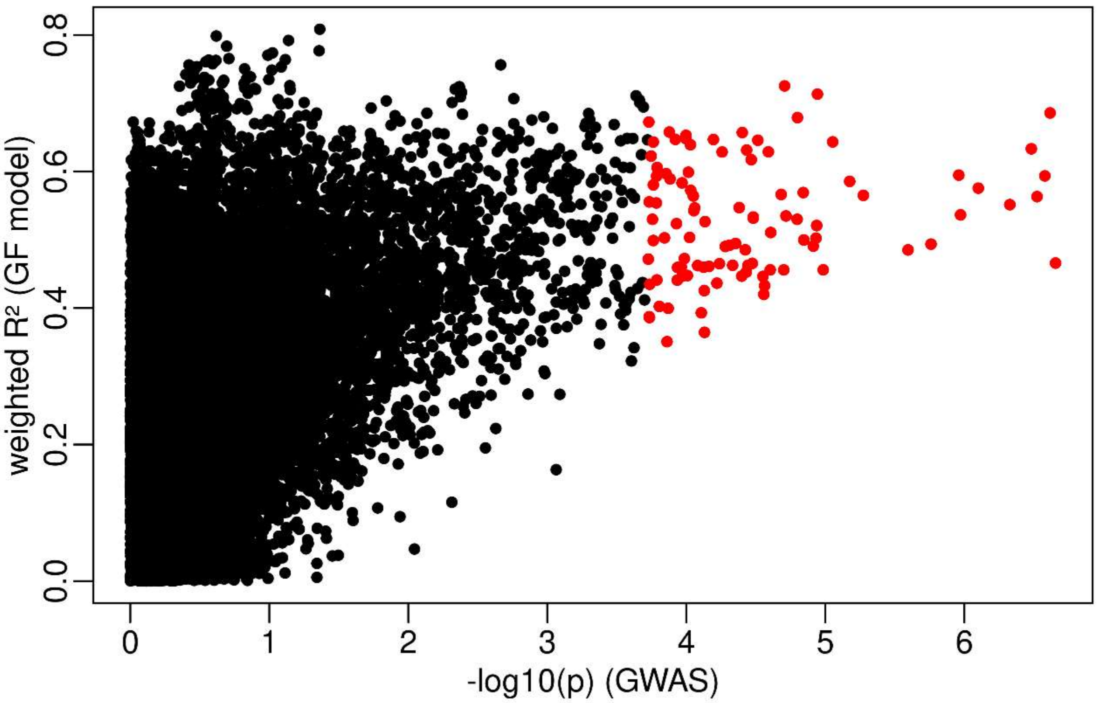

Supplementary Figure 6. **SNPs found associated with flowering time have high correlation in the gradient forest model.**

We plot the SNPs *p*-value of the GWAS association with flowering time ($-\log_{10}(p)$) against the weighted R^2 value of each SNPs provided by the gradient forest modelling. This R^2 value assesses the relative importance of predictor variables in the GF model.

The red dots correspond to the SNPs significantly associated to flowering time with a 5% FDR threshold.

The 103 SNPs associated with flowering time exhibit a two time higher correlation in the GF model (mean(R^2) = 0.53) compared to all the considered SNPs (mean(R^2) = 0.28, Wilcoxon rank test, *p*-val. $<2.10^{-16}$). We obtained exactly the same values considering only the 83 SNPs found in association with flowering time for a number of latent factors of K=6 and K=7 in the GWAS analyses.

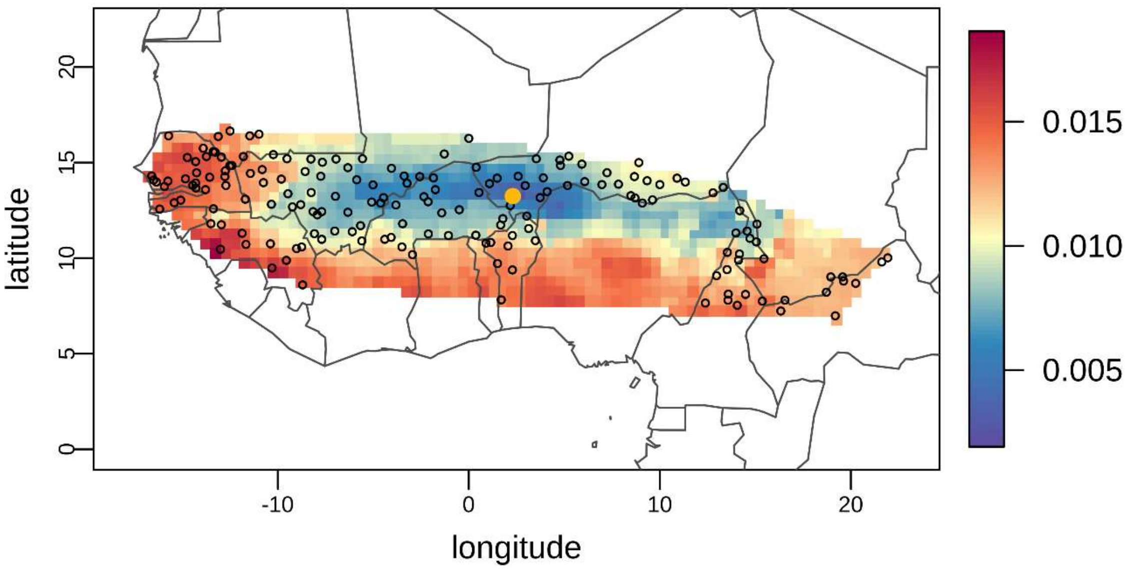

Supplementary Figure 7. **Projection of the genomic vulnerability of the common garden experiment.**

We used spatial climate contrast to assess vulnerability at the experimental site. The climate at the location of the field experiment in Sadoré, Niger was used to represent the future climate of the whole map. Genomic vulnerability of pearl millet landraces was consequently predicted under the climate condition prevailing at the location of origin of the landrace and the climate of the field experiment. Under the field experiment conditions, genomic vulnerability increased with the geographic distance to Sadoré. This increase is higher for latitude than for longitude, which is consistent with the higher latitudinal climate variation generally observed in climate data. Circles correspond to the location of the landraces sampled to evaluate yield related traits in the common garden experiment that were subsequently used to link genomic vulnerability and yield (Fig. S8).

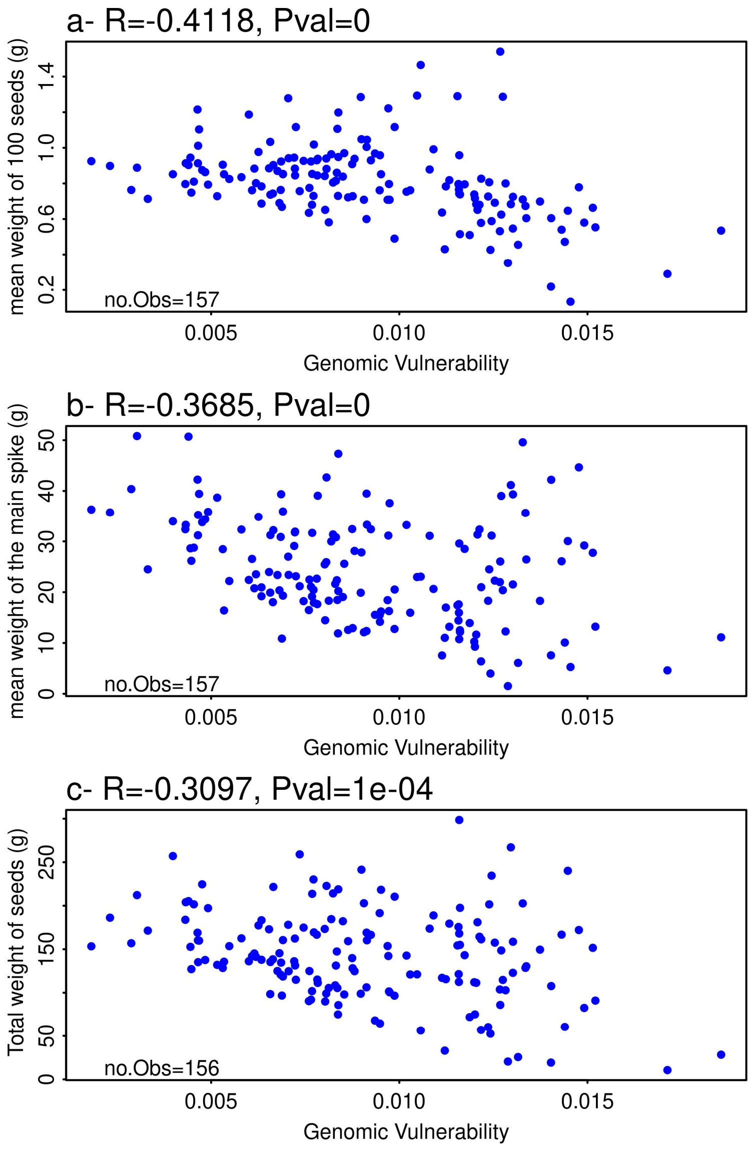

Supplementary Figure 8. **Genomic vulnerability is associated with yield related traits**

Correlations between the genomic vulnerability of the pearl millet landraces under the climate conditions at Sadoré and the yield related traits measured in the common garden experiment in Sadoré (Niger). **a** Correlation between the genomic vulnerability and the mean 100-seed weight; **b** Correlation between the genomic vulnerability and the mean weight of the main spike; **c** Correlation between the genomic vulnerability and the mean weight of all the seeds.

For the mean weight of seeds on the main spike and the weight of seeds per plant, these correlations are higher than the correlations with the geographic distance from the origin of the variety (*r*(Pearson)=-0.283 and *r*(Pearson)= 0.311 respectively). This result suggests that the climatic distance explains the genomic structuration linked with productivity better than a simple geographic distance.

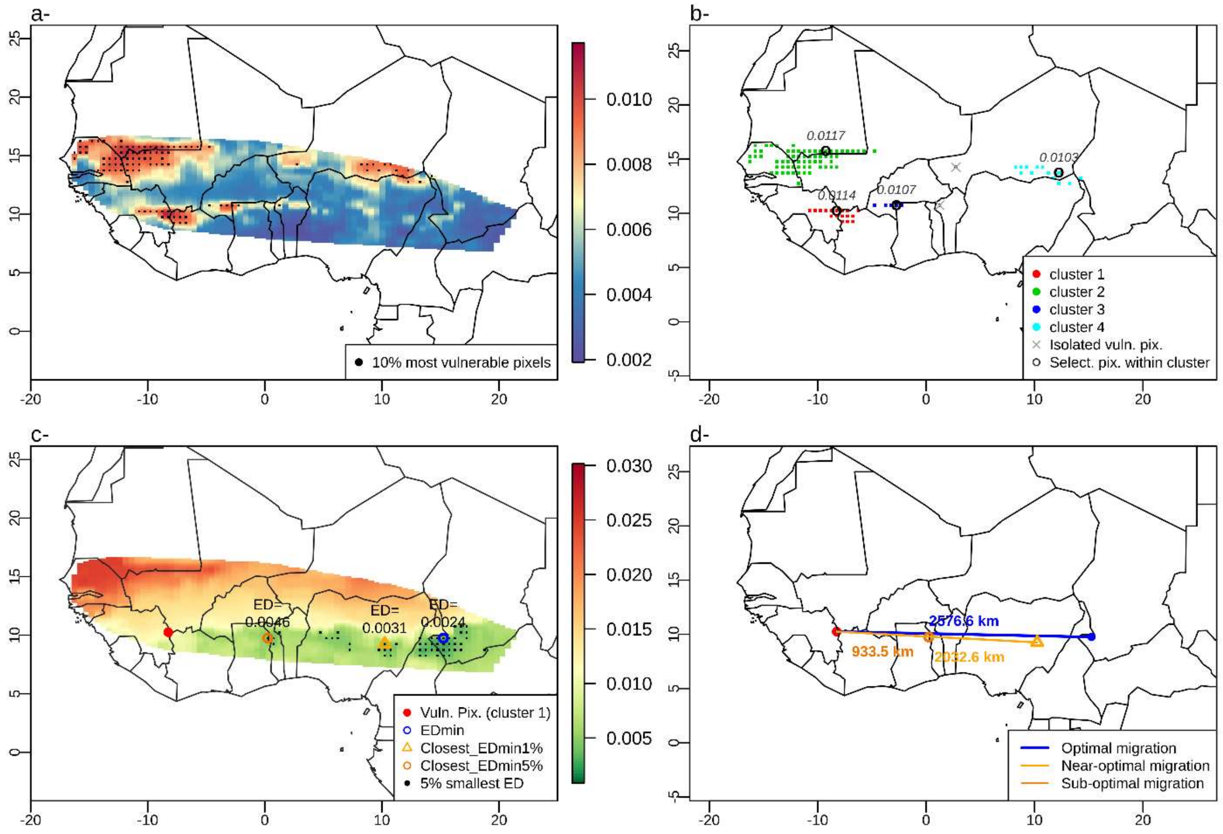

Supplementary Figure 9. **Methodology for assessing assisted varietal migration.**

The main steps of assisted migration are illustrated in detail using historical and future climate projections of the bcc-csm1-1 climate model (Beijing Climate Center).

a *Map of the genomic vulnerability for the climate model considered.* We assessed the genomic vulnerability of the climate model considered for the year 2050 in the 8.5 emission scenario. The red areas correspond to the most vulnerable areas for the cultivation of pearl millet landraces under projected future climate conditions. Dark stippling indicates the 10% most vulnerable pixels among the 1,041 total pixels covering the cultivation area, such as climate data and hence genomic vulnerability at a pixel resolution of 0.5° x 0.5°.

b *Identification of vulnerable areas.* The most vulnerable pixels tended to be grouped in vulnerable areas on the map. To summarize this patchy organization, we used a clustering approach based on the geographical distance between vulnerable pixels to group closely packed pixels and hence to correctly identify the most vulnerable areas. In the example illustrated here, four distinct clusters are visible. Isolated vulnerable pixels (grey crosses) were not retained for the following analysis. Within each cluster, we selected the most vulnerable pixel to be used as the reference of the cluster for migration assessment.

c *Map of the genomic vulnerability of currently cultivated landraces under future climate conditions forecasted for a vulnerable area.* For each vulnerable area identified, we predicted that migration would enable mitigation of future climate impact. To give an example, here, we assessed migration for the vulnerable area identified as cluster 1 (plain red circle). We measured the genomic vulnerability of currently cultivated landraces under the future climate conditions forecast for this vulnerable area. This was obtained from the Euclidian distance between the genomic composition under current climate conditions throughout the cultivation area and the genomic composition under the specific future climate conditions forecast for the vulnerable area. We were then able to identify red areas corresponding to regions in which landraces will not be adapted these specific future climate conditions, and green areas in which landraces will be found that are adapted to this specific future climate. The blue circle indicates the location with the shortest Euclidian distance (EDmin) in which current cultivated landraces may be the best adapted to the future climate conditions forecast in the vulnerable area concerned. The EDmin value (EDmin=0.0024 in this example) corresponds to the genomic vulnerability of migrated landraces and measures the migration load of this optimal migration. The orange triangle corresponds to the location of the pixel located closest to the vulnerable area among the 1% less vulnerable pixels for a near-optimal migration (Closest_EDmin1%=0.0031). The orange circle corresponds to the location of a sub-optimal migration corresponding to the 5% less vulnerable pixels (Closest_EDmin 5%=0.0045). The analysis was conducted vulnerable area by vulnerable area, so in this particular climate model, 4 migrations are assessed.

d *Migration trajectories and distances depending on the type of migration.* The migration analysis was performed for all climate models and all vulnerable area identified in each model. The geographic migration distances were measured under optimal (blue trajectory), near-optimal (light orange) and sub-optimal (dark orange) conditions.

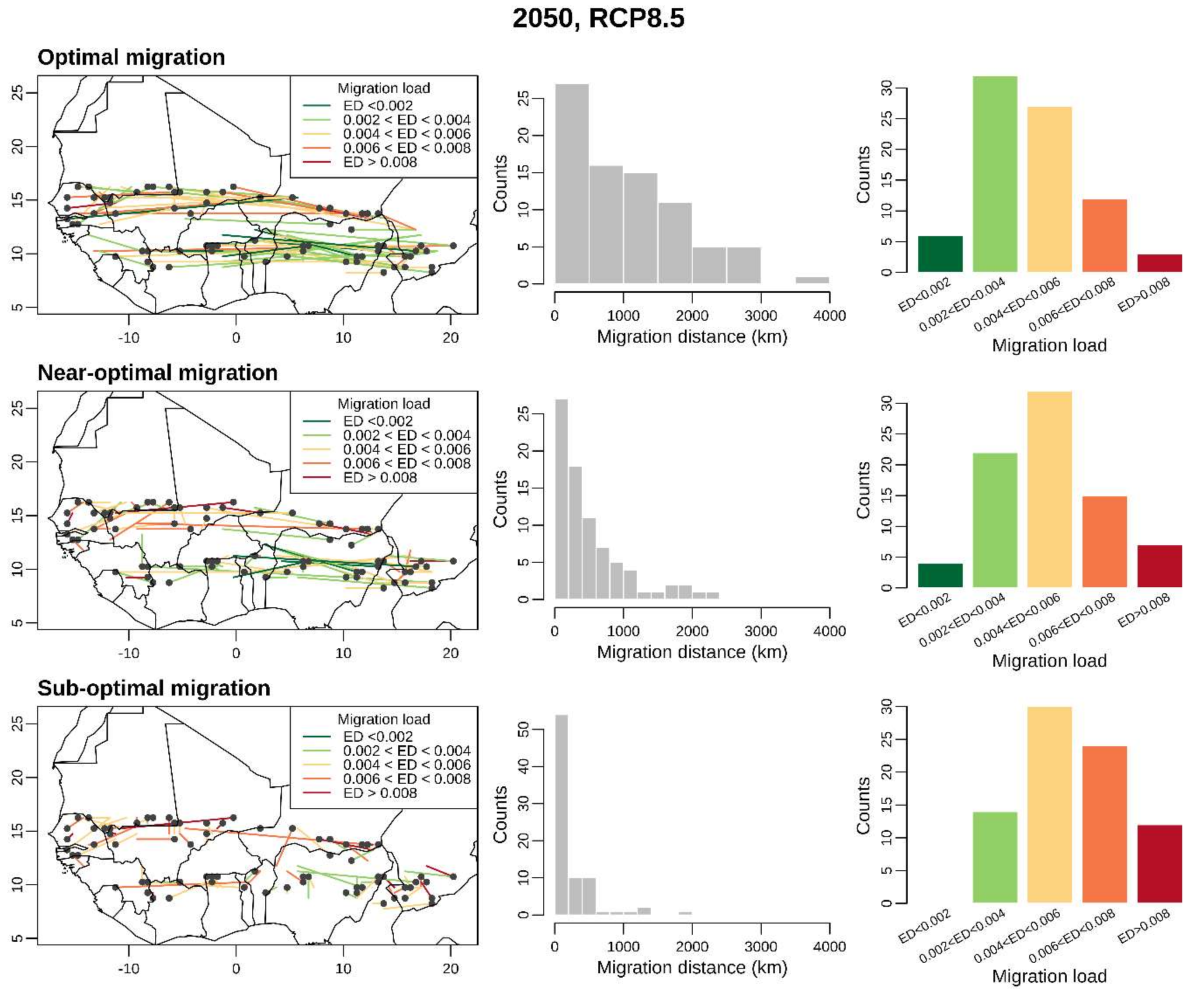

Supplementary Figure 10. **Assisted migration of pearl millet varieties for adaptation to future climate by 2050 under the RCP8.5 scenario.**

Optimal migration refers to the migration scenario that minimizes the future genomic vulnerability of the migrated varieties. Near-optimal and sub-optimal migration correspond to migration scenarios that minimize the geographic migration distance among the 1% and 5% less vulnerable migrated varieties in the future.

Three plots were drawn for each migration scenario:

- a map of the migration trajectories. Colored lines link one of the most vulnerable locations (black dots) to the location selected for the migration of varieties to mitigate future genomic vulnerability (right panel).
- the migration distance distribution (central panel)
- the migration load distribution (left panel). The migration load refers to the genomic vulnerability of landraces after migration under future climate projections. High migration load values indicate migrations that rely on migrated varieties that may not be perfectly adapted as no other varieties that are better adapted to future climate condition exist in the cultivation area.

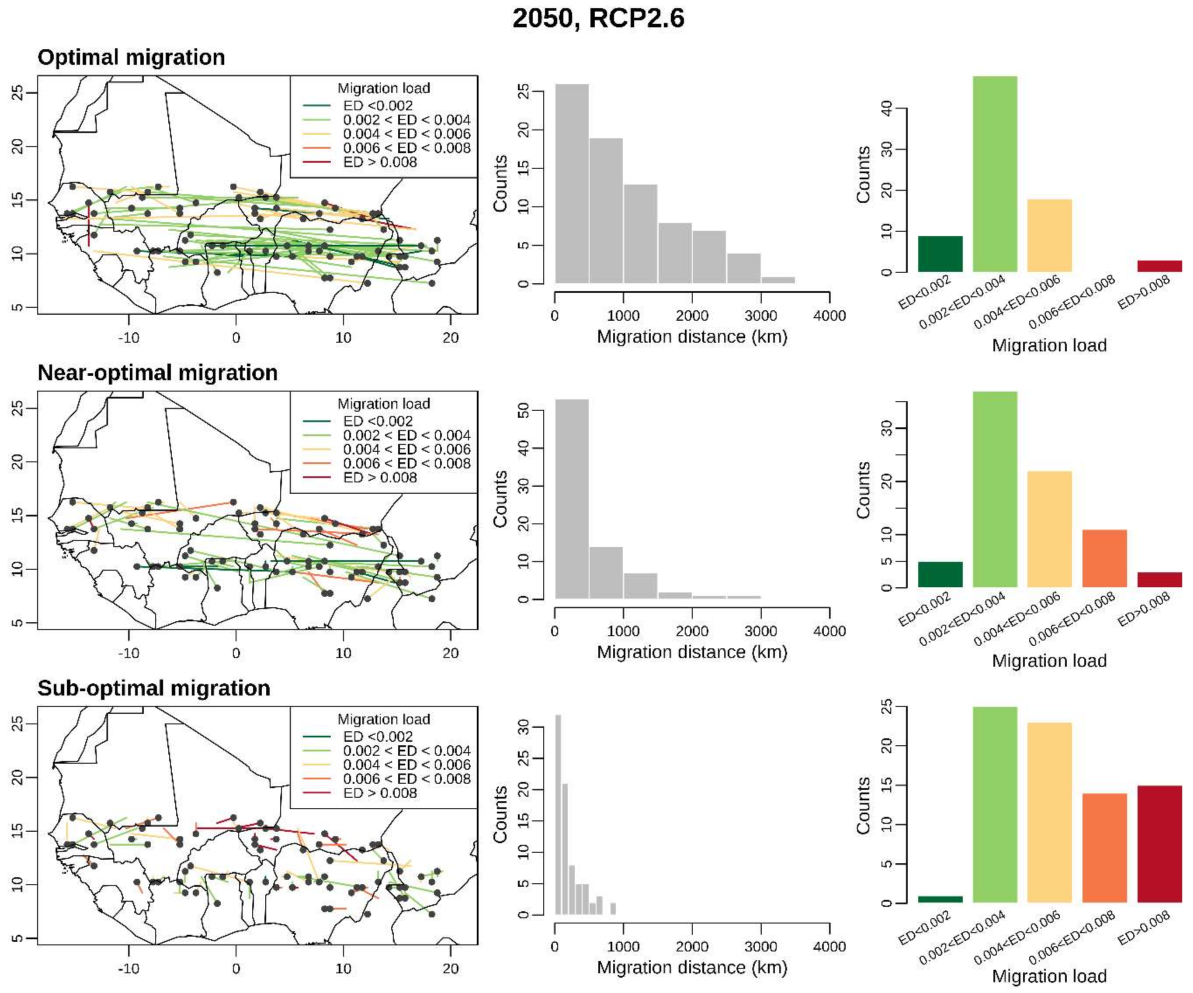

Supplementary Figure 11. **Assisted migration of pearl millet varieties for adaptation to future climate by the 2050 horizon under the RCP2.6 scenario.**

(same legend as Supplementary Figure 10)

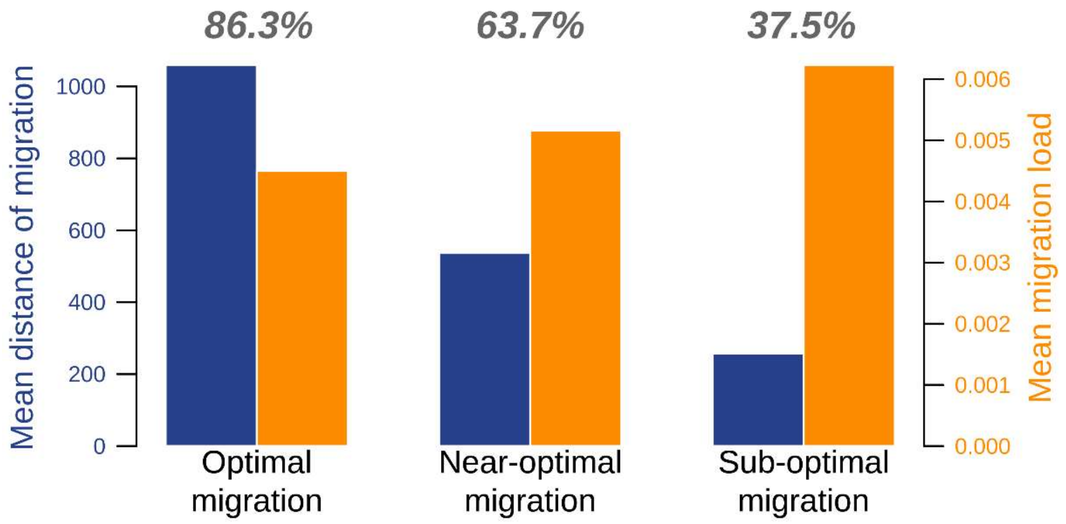

Supplementary Figure 12. **Migration distance and migration load as a function of the migration scenario**

Optimal migration refers to the migration scenario that minimizes the future genomic vulnerability of the migrated varieties. Near-optimal and sub-optimal migration correspond to the migration scenario that minimizes the geographic migration distance among the 1% and 5% less vulnerable migrated varieties in the future. Grey numbers in italics indicate the percentage of inter-country migration among all the migration trajectories.

Supplementary Table 1. **List of the climate metrics used for gradient forest modelling**

**Category**	**metrics**	**Description**
Onset	Onset	Local Agronomic Monsoon Onset Date: the first rainy day (> 1 mm) of two consecutive rainy days (with total precipitation > 20 mm) and no 7-day dry spell (< 5 mm) of rainfall during the subsequent 20 days
PR* (Precipitation)	cumul	Cumul of rain for the selected period (mm)
	wetspell	Number of periods with rainfall longer than 10 consecutive days
	dryspell	Number of periods with no rainfall during more than 6 days for the selected period
	intensity	Mean daily rainfall on rainy days (mm.day^{-1})
	max3	Maximum rainfall total in a 3-day period (mm)
	max5	Maximum rainfall total in a 5-day period (mm)
	no_day_rain	Number of rainy days (>1mm day^{-1}) during the selected period
	no_day_rain_30	Number of rainy days > 30 mm during the selected period
	no_day_rain_50	Number of rainy days > 50 mm during the selected period
TAS* (mean near-surface air temperature)	mean	Mean daily near-surface temperature (°C)
	min	Minimum of mean daily temperature for the selected period (°C)
	max	Maximum of mean daily temperature for the selected period (°C)
	no_day_T30	Number of days with a maximum daily temperature > 30°C during the selected period
TAS-MIN* (near-surface minimum air temperature)	mean	Mean of the minimum registered temperature during the selected period (°C)
	min	Minimum value of the minimum registered temperature during the selected period (°C)
	max	Maximum value of the minimum registered temperature during the selected period (°C)
	no_day_T20	Number of days for which the minimum temperature exeed 20°C during the selected period
	no_day_T30	Number of days for which the minimum temperature exeed 30°C during the selected period
TAS-MAX* (near-surface maximum air temperature)	mean	Mean of the maximum registered temperature during the selected period (°C)
	min	Minimum value of the maximum registered temperature during the selected period (°C)
	max	Maximum value of the maximum registered temperature during the selected period (°C)
	no_day_T30	Number of days for which the maximum temperature exeed 30°C during the selected period
	no_day_T40	Number of days for which the maximum temperature exeed 40°C during the selected period
RSDS* (Surface Downwelling Shortwave Radiation)	cumul	Average surface downwelling shortwave radiation (W.m^2) during the selected period
	min	Minimum Surface Downwelling Shortwave Radiation (W.m^2)
	max	Average Surface Downwelling Shortwave Radiation (W.m^2)

* Climate metrics obtained for Onset+30 (period between the monsoon onset and 30 days after the monsoon onset), Onset+60, Onset+90, Onset+120, Onset+150, Onset+180

Supplementary Table 2. **List of the climate models used for gradient forest modelling**

**Modelling center**	**CMIP5 models**
Beijing Climate Center (China)	bcc-csm1-1
	bcc-csm1-1-m
Global Change and Earth System Science, Beijing Normal University (China)	BNU-ESM
Canadian Centre for Climate Modelling and Analysis (Canada)	CanESM2
Centre National de Recherches Météorologiques - Centre Européen de Recherche et Formation Avancée en Calcul Scientifique (France)	CNRM-CM5
National Oceanographic and Atmospheric Administration, Geophysical Fluid Dynamics Laboratory (United States)	GFDL-CM3
	GFDL-ESM2G
	GFDL-ESM2M
Met Office Hadley Centre (United Kingdom)	HadGEM2-ES
	HadGEM2-AO
Institut Pierre-Simon Laplace (France)	IPSL-CM5A-LR
	IPSL-CM5A-MR
Model for Interdisciplinary Research on Climate (Japan)	MIROC5
	MIROC-ESM
Max-Planck-Institut für Meteorologie (Germany)	MPI-ESM-LR
	MPI-ESM-MR
Meteorological Research Institute (Japan)	MRI-CGCM3

Supplementary Table 3. **Gradient forest models results**

model	No.SNP $R^2>0$	Five most important climate predictors*
MIROC-ESM	14,660	RSDS_min_60, RSDS_min_30, Monsoon_Onset, RSDS_max_30, TMAX_min_120
CNRM-CM5	14,719	Monsoon_Onset, RSDS_min_30, RSDS_min_60, RSDS_min_90, RSDS_max_30
GFDL-CM3	14,683	RSDS_min_30, RSDS_min_60, Monsoon_Onset, RSDS_max_30, RSDS_cumul_30
MPI-ESM-LR	14,718	RSDS_min_30, PR_intensity_30, RSDS_min_60, RSDS_max_30, RSDS_min_90
MPI-ESM-MR	14,642	RSDS_min_30, RSDS_min_60, RSDS_max_30, PR_intensity_60, PR_intensity_30
MRI-CGCM3	14544	RSDS_min_60, RSDS_min_30, RSDS_min_90, RSDS_max_30, PR_intensity_30
BNU-ESM	14,628	RSDS_min_30, RSDS_min_60, RSDS_max_30, PR_intensity_60, Monsoon_Onset
CanESM2	14,650	Monsoon_Onset, RSDS_min_30, RSDS_min_60, RSDS_max_30, PR_intensity_60
HadGEM2-ES	14,687	RSDS_min_60, PR_intensity_60, RSDS_min_30, RSDS_min_90, RSDS_max_30
IPSL-CM5A-LR	14,660	PR_intensity_60, RSDS_min_60, PR_intensity_30, RSDS_min_30, RSDS_max_30
IPSL-CM5A-MR	14,631	RSDS_min_30, RSDS_min_60, RSDS_max_30, RSDS_min_120, PR_intensity_30
GFDL-ESM2G	14,587	RSDS_min_30, RSDS_min_60, PR_intensity_30, PR_intensity_60, RSDS_max_30
GFDL-ESM2M	14,681	RSDS_min_60, Monsoon_Onset, RSDS_min_30, RSDS_min_90, RSDS_max_30
bcc-csm1-1	14,757	RSDS_min_30, RSDS_min_60, Monsoon_Onset, PR_intensity_30, RSDS_min_90
bcc-csm1-1-m	14,746	RSDS_min_60, RSDS_min_30, RSDS_min_90, PR_intensity_60, RSDS_max_30
HadGEM2-AO	14,717	RSDS_min_60, RSDS_min_30, RSDS_min_90, PR_intensity_60, RSDS_max_30
MIROC5	14,725	RSDS_min_60, Monsoon_Onset, RSDS_min_30, PR_intensity_60, RSDS_max_30
Observed Climate (EWEMBI dataset)	14,557	RSDS_max_30, PR_intensity_30, Monsoon_Onset, PR_intensity_60, RSDS_min_60

*Except for the monsoon onset, the climate metrics are coded as follow: the first letters correspond to the category of the climate variable (*i.e.* PR: Precipitation, RSDS: Surface Downwelling Shortwave Radiation, see details in Table S3), followed by the reference of the metric (*i.e.* intensity, min, max …, see details in Table S3), followed by the number of day after the monsoon onset (30, 60, 90; 120, 150, 180).
