# Supplementary material for: Pearl millet genomic vulnerability to climate change in West Africa highlights the need for regional collaboration
Source: Nat Commun. 2020 Oct 19;11:5274. doi: 10.1038/s41467-020-19066-4 (PMC7573578; doi:10.1038/s41467-020-19066-4)
Supplement: Supplementary file 3 — Description of Additional Supplementary Files [file 41467_2020_19066_MOESM3_ESM.pdf]

Description of Additional Supplementary Files

File Name: Supplementary Data 1

Description: List of landraces and passeport data

File Name: Supplementary Data 2

Description: Landraces exom coverage estimation for pool-sequencing

File Name: Supplementary Data 3

Description: Annotation of the candidate genes associated with flowering time
