# Supplementary material for: Pearl millet genomic vulnerability to climate change in West Africa highlights the need for regional collaboration
Source: Nat Commun. 2020 Oct 19;11:5274. doi: 10.1038/s41467-020-19066-4 (PMC7573578; doi:10.1038/s41467-020-19066-4)
Supplement: Supplementary file 7 — Reporting Summary [file 41467_2020_19066_MOESM7_ESM.pdf]

nature research

Corresponding author(s): Rhoné Bénédicte
Vigouroux Yves

Last updated by author(s): Sep 17, 2020

Reporting Summary

Nature Research wishes to improve the reproducibility of the work that we publish. This form provides structure for consistency and transparency in reporting. For further information on Nature Research policies, see our Editorial Policies and the Editorial Policy Checklist.

Statistics

For all statistical analyses, confirm that the following items are present in the figure legend, table legend, main text, or Methods section.

n/a	Confirmed	
☐	☒	The exact sample size (n) for each experimental group/condition, given as a discrete number and unit of measurement
☐	☒	A statement on whether measurements were taken from distinct samples or whether the same sample was measured repeatedly
☐	☒	The statistical test(s) used AND whether they are one- or two-sided<br>*Only common tests should be described solely by name; describe more complex techniques in the Methods section.*
☐	☒	A description of all covariates tested
☐	☒	A description of any assumptions or corrections, such as tests of normality and adjustment for multiple comparisons
☐	☒	A full description of the statistical parameters including central tendency (e.g. means) or other basic estimates (e.g. regression coefficient) AND variation (e.g. standard deviation) or associated estimates of uncertainty (e.g. confidence intervals)
☐	☒	For null hypothesis testing, the test statistic (e.g. F, t, r) with confidence intervals, effect sizes, degrees of freedom and P value noted<br>*Give P values as exact values whenever suitable.*
☒	☐	For Bayesian analysis, information on the choice of priors and Markov chain Monte Carlo settings
☒	☐	For hierarchical and complex designs, identification of the appropriate level for tests and full reporting of outcomes
☐	☒	Estimates of effect sizes (e.g. Cohen's d, Pearson's r), indicating how they were calculated

Our web collection on statistics for biologists contains articles on many of the points above.

Software and code

Policy information about availability of computer code

Data collection	No software was used for data collection
Data analysis	The scripts used for the bioinformatic analysis are available in ZENODO (https://zenodo.org/record/3970815). The software used were Cutadapt v1.10 , BWA v0.7.2, SAMtools v1.1, QualiMap (v2.2), GATK v3.7, bedtools (v2.27.1).<br>We conducted the data analysis and drew figures using the R packages dbscan (v1.1-3), factoextra (v1.0.5), fields (v9.8-1), geosphere (v1.5-7), ggmap (v3.0.0), ggplot2 (v3.1.1), gradientForest (v0.1-17), lfmm (v1.0), locfit (v1.5-9.1), q-value (v2.18.0) and custom R code in R version 3.5.3. All the R code are available in ZENODO (https://doi.org/10.5281/zenodo.3970815)

For manuscripts utilizing custom algorithms or software that are central to the research but not yet described in published literature, software must be made available to editors and reviewers. We strongly encourage code deposition in a community repository (e.g. GitHub). See the Nature Research guidelines for submitting code & software for further information.

Data

Policy information about availability of data

All manuscripts must include a data availability statement. This statement should provide the following information, where applicable:

- Accession codes, unique identifiers, or web links for publicly available datasets
- A list of figures that have associated raw data
- A description of any restrictions on data availability

All the biological data that support the findings of this study are available with no restriction. The raw sequencing data is available in the NCBI Sequence Read Archive (SRA) database with links to Bioproject accession number PRJNA422966 (https://www.ncbi.nlm.nih.gov/bioproject/). The SNPs allele frequency and the phenotypic data generated in the study are available in ZENODO (https://doi.org/10.5281/zenodo.3970815).
The EWMBI climate dataset is available in the GFZ Data Services repository with the identifier https://doi.org/10.5880/pik.2016.004. The CDF-t bias-corrected

CMIP5 climate data over Africa are available at http://amma2050.ipsl.upmc.fr/. To access the data, users must contact the lead author at moflod@locean-ipsl.upmc.fr. The sample of those climate datasets extracted for this study and used in the analysis is available in ZENODO (https://doi.org/10.5281/zenodo.3970815).

Field-specific reporting

Please select the one below that is the best fit for your research. If you are not sure, read the appropriate sections before making your selection.

☐ Life sciences ☐ Behavioural & social sciences ☒ Ecological, evolutionary & environmental sciences

For a reference copy of the document with all sections, see nature.com/documents/nr-reporting-summary-flat.pdf

Ecological, evolutionary & environmental sciences study design

All studies must disclose on these points even when the disclosure is negative.

Study description	We analyzed the genomic diversity of 173 landraces collected in West Africa together with a extensive climate dataset composed of metrics of agronomical importance. Using a gradient forest approach , we model current genomic-environment relationship to create landscape predictions in the future based on 17 models projection of climate change and two scenario of CO2 gas emission.
Research sample	The research sample consistes in 173 landraces of pearl millet (Cenchrus americanus (L.)). Our sample consitutes an exhaustive representation of the diversity of the local varieties cultivated across West-africa in family farming systems. Pearl millet is a staple food for more than 90 million people in the arid and semi-arid tropical regions of Africa and Asia.<br>The climate datasets consisted in observed
Sampling strategy	Landraces' sample size was defined so that one landrace is collected within each pixel of 0.5° x 0.5° of the climate data. A total of 173 landraces were chosen to cover the pearl millet cultivation area in West Africa among the available varieties at the IRD collection. It was not possible to collect landraces from Nigeria due to the geo-political context.<br>For the pool-sequencing experiment, a total of 100 individuals from each of the 173 landraces were used to accurately estimate SNPs allele frequency of each landrace from allele counts.
Data collection	DNA extractions were performed at IRD in Montpellier, France by M Couderc & A Dequincey<br>DNA banks and DNA captures were performed at IRD in Montpellier by M Couderc, C Mariac<br>Sequencing was performed by the NOVOGEN company (China) using four Illumina sequencing lanes on a HiSeq2500<br>Bioinformatic analysis and SNP calling were performed on the IRD itrop HPC cluster (South Green Platform) by B Rhoné in collaboration with Y Vigouroux, A Barnaud and C Mariac<br>Phenotyping was done at Sadoré,Niger and supervized by Y Vigouroux, A Barnaud, C Berthouly-Salazar and L Zekraoui
Timing and spatial scale	The landraces were collected by the Institut de la Recherche pour le Développement (IRD) on dedicated missions conducted between 1974 and 1989 in villages in West-Africa within the area of culture of pearl millet extending over 3 million km2. The collect missions were organized at the maturation date of the pearl millet, varying between october and february depending on the country and latitude.<br>Sequencing and SNPs allele frequency data acquisition were performed in 2018.<br>Plant phenotyping was done during the rainy season, corresponding to the usual period for pearl millet cultivation in Sadoré (Niger), in 2016 and 2017 to take into account for inter-annual climate variabilities.
Data exclusions	In the pool-seq experiment, a landrace (PE05487) has been sequenced twice to test for reproducibility. We only include the replication with the higher number of reads in the final dataset to estimate the SNPs allele frequency of this landrace.<br>Raw SNPs were filtered out using the following criteria: bi-allelic SNP, depth coverage (DP) > 10 and < 250 per accession, less than three SNPs in a window of 5 bp, frequency of the alternate allele to be called as a SNP (AF > 0.003 corresponding to a minimum count of five reads with the alternate allele throughout the dataset). For each accession, SNPs with a total read count of less than 20 reads were set to NA. Finally, only the 138,948 SNPs with complete data were considered in the final SNPs set to perform the PCA analysis.<br>For the gradient forest analysis, we only considered the 16,632 SNPs with a minor allele frequency > 10% following Bay et al (2018), both to limit computation time and because rare alleles are more likely to yield false positives.
Reproducibility	Field trials were repeated 6 times (two distinct trials in 2016 and four trials in 2017) and show consistency.<br>For sequencing, we include the same landrace PE05487 in different batch of sequencing, and calculated correlation of allele frequency across three sequencing replications (r(Pearson)=0.91 to 0.96, n=5 275).<br>Gradient forest analysis linking genetic and climate data were done with and without correlated climatic variables and conducted to similar partern of genomic vulnerability assessment.<br>Gradient forest is a machine-learning modeling approach based on regression trees linking allele frequencies with climate data observed at the landraces origin. To construct the gradient-forest models, 500 regression trees per SNP were generated with boostrap of the observations.
Randomization	Field trials were done by fully randomizing varieties and using three full blocks. Two repetitions were done in 2016 and four repetitions in 2017.
Blinding	Not relevant here as the field trials were performed by technicians not aware of the origine of the landraces in a randomized experiment.

Did the study involve field work? [x] Yes [ ] No

Field work, collection and transport

Field conditions	The phenotypic dataset was obtained during the rainy season in Sadoré, Niger in 2016 and 2017. The experiment was done under natural rainfall supply with irrigation if needed. The mean mensual temperatures at the experiment location is comprised between 28 and 29°C at the rainy season (July-September), the mean mensual precipitation is comprised between 77 to 152 mm at the same period.
Location	Field trials were performed at the International Crops Research Institute for the Semi-Arid Tropics (ICRISAT) field station in Sadoré, Niger (Lat. 13.2375, Long. 2.2797)
Access & import/export	The landraces of cultivated pearl millet were collected between 1974 and 1989 in West-Africa by IRD researchers. These emergency collection of germplasm organized by the Food and Agriculture Organisation of the United Nations (FAO) were performed by subcontract to the IRD (previously ORSTOM) and funded by the United Nations Environment Program (UNEP). The samples were left at the disposal of the country of origin and both stored at the International Crops Research Institute for the Semi-Arid Tropics (ICRSAT) and IRD facilities in Montpellier.
Disturbance	The experiment was done on a field experimental station, no disturbance was identified.

Reporting for specific materials, systems and methods

We require information from authors about some types of materials, experimental systems and methods used in many studies. Here, indicate whether each material, system or method listed is relevant to your study. If you are not sure if a list item applies to your research, read the appropriate section before selecting a response.

Materials & experimental systems

n/a	Involved in the study
[x]	[ ] Antibodies
[x]	[ ] Eukaryotic cell lines
[x]	[ ] Palaeontology and archaeology
[x]	[ ] Animals and other organisms
[x]	[ ] Human research participants
[x]	[ ] Clinical data
[x]	[ ] Dual use research of concern

Methods

n/a	Involved in the study
[x]	[ ] ChIP-seq
[x]	[ ] Flow cytometry
[x]	[ ] MRI-based neuroimaging
